# Relationship of Platelet Counts and Inflammatory Markers to 30-Day Mortality Risk in Patients with Acute Type A Aortic Dissection

**DOI:** 10.1155/2020/1057496

**Published:** 2020-04-21

**Authors:** Yiping Chen, Yanjuan Lin, Haoruo Zhang, Yanchun Peng, Sailan Li, Xizhen Huang

**Affiliations:** ^1^Department of Nursing, Fujian Medical University Union Hospital, Fuzhou, China; ^2^Fujian Medical University, Fuzhou, China; ^3^Department of Cardiac Surgery, Fujian Medical University Union Hospital, Fuzhou, China

## Abstract

Markers of prothrombotic state and inflammation are associated with the prognosis of patients with acute type A aortic dissection (AAAD). However, it is unclear that the relationship between these biomarkers and their combined impact on risk stratification. The present study evaluated the prognostic value of platelet counts, lymphocyte to neutrophil ratio (LNR), and lymphocyte to monocyte ratio (LMR), alone and in combination. A retrospective analysis of clinical data of 744 AAAD patients was conducted to identify whether these biomarkers were related to the 30-day mortality risk. A Kaplan-Meier analysis and log-rank test were used to compare survival between groups. A Cox hazard regression multivariable analysis was performed for 30-day mortality. Individual biomarker (platelet count, LNR, or LMR) was unable to predict 30-day mortality. However, combinations of all three biomarkers provided additive predictive value over either marker alone, the receiver operating characteristic (ROC) model had a prediction probability of 0.739 when platelet counts, LNR, and LMR were included. Cox hazard regression multivariable analysis showed that combinations of all three biomarkers were the strongest predictor of 30-day mortality (*p* < 0.021). Combined with these three easily measurable biomarkers at admission, they could help identify AAAD patients with a high risk of 30-day mortality.

## 1. Introduction

Acute type A aortic dissection (AAAD) is a life-threatening cardiovascular emergency with high rates of early morbidity and mortality after the onset of symptom [1]. Both inflammation and hemostatic play important roles in the pathogenesis of AAAD [2]. Various inflammatory and hemostatic biomarkers have attracted more and more attention as prognostic indicators in AAAD patients [3–6]. Risk stratification using such biomarkers can help predict patients' outcome which is important for the healthcare providers to deliver appropriate therapeutic management [7].

Both lymphocyte to monocyte ratio (LMR) and lymphocyte to neutrophil ratio (LNR), obtained from routine blood tests at admission, are readily available inflammatory biomarkers [8, 9]. Previous studies have found that LMR and LNR both were good predictors of worse outcomes in patients with AAAD [10, 11]. However, the comparative predictive values of LMR and LNR are unclear. Moreover, studies have demonstrated that platelet was a specific biomarker of the activation of hemostatic pathways [12]. Decreased platelet counts and platelet dysfunction have been observed in patients with AAAD [13, 14]. Low admission platelet counts may be a strong predictor of increased in-hospital mortality in AAAD patients with or without surgical intervention [5].

Although the individual prognostic ability of LMR, LNR, and platelet counts in AAAD has been studied extensively, no study has investigated their combined predictive value. Therefore, in this study with a relatively large sample size, we aimed to assess the predictive value of these biomarkers, either alone or in combination, for the prediction of 30-day mortality in patients with AAAD.

## 2. Methods

### 2.1. Study Design and Patients

From June 2012 to June 2019, consecutive patients with AAAD who admitted to the emergency center of Fujian Province Heart Medical Center were enrolled in our study. The diagnosis of AAAD was confirmed by computed tomographic angiography (CTA) or magnetic resonance angiography (MRA). Patients were excluded if they lost to follow-up within 30 days or lacked of clinical data. All patients with chronic dissections or previous operations were also excluded. The study protocol was approved by the Ethics Committee of Fujian Medical University Union Hospital.

### 2.2. Data Collection

Both baseline characteristics data and clinical characteristics were recorded in our medical system. Baseline characteristics included sex, age, body mass index (BMI), and medical history including coronary artery disease, diabetes mellitus, hypertension, drinking status, and smoking status. Clinical features included baseline vital signs, including systolic/diastolic blood pressure and heart rate at admission, laboratory inspection at admission and hospital management (surgical intervention or medical therapy). The laboratory results were obtained using the patients' first venous blood samples taken on admission to the hospital, and the samples were measured by Shenzhen Mindray BC-5800 blood cell analyzer. The principles and strategies of surgical techniques were determined by experienced surgeons in the Fujian Province Heart Medical Center. Patients' in-hospital outcomes were gathered from medical records. After discharge, patients' follow-up was performed through an outpatient visit or a telephone every 1 month.

### 2.3. Statistical Analysis

Normally distributed data were shown as the mean values ± standard deviation, and nonnormally distributed data were shown as median (interquartile range). Categorical variables were presented in the form of frequencies and percentages. The chi-square test or Fisher's exact test was used to compare the categorical variables between the two groups. Correlation analysis was conducted by the Pearson method. The optimal cut-off values of platelet count, LNR, and LMR were determined using the receiver operating characteristic (ROC) curve. A Kaplan-Meier analysis and log-rank test were used to compare survival between groups. A Cox hazard regression multivariable analysis was performed for 30-day mortality including variables with *p* value of less than 0.05 in univariate analysis. Analyses were performed with SPSS 24.0, a *p* value <0.05 was considered as statistically significant.

## 3. Results

### 3.1. Clinical Features of AAAD Patients

**Figure 1:**
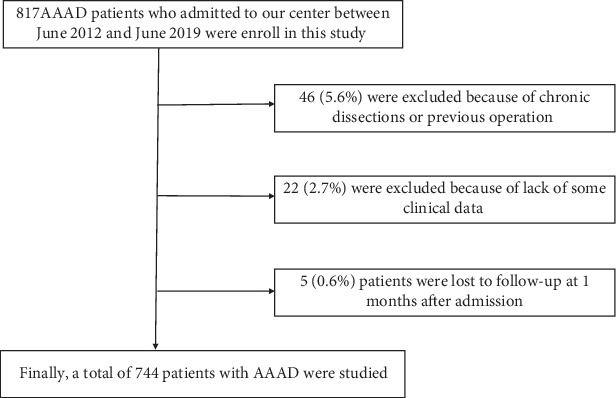
Patient flow chart of the cohort. AAAD: acute type A aortic dissection.

We identified 817 patients who were admitted to our center between June 2012 and June 2019. Of these patients, 46 (5.6%) were excluded because of chronic dissections or previous operation, 22 (2.7%) were excluded because of lacking of some clinical data, and 5 (0.6%) patients were lost to follow-up at 1 month after admission. Finally, a total of 744 patients with AAAD were studied (Figure 1), of which 571 were male patients.  Table 1 lists baseline characteristics according to the 30-day mortality. Older age, smoker, lower pulse pressure, higher D-dimer and higher serum creatinine were associated with higher 30-day mortality, while patients who received the surgical intervention had a lower risk of 30-day mortality.

### 3.2. Individual Predictive Values of Platelet Counts, LNR, and LMR

**Figure 2:**
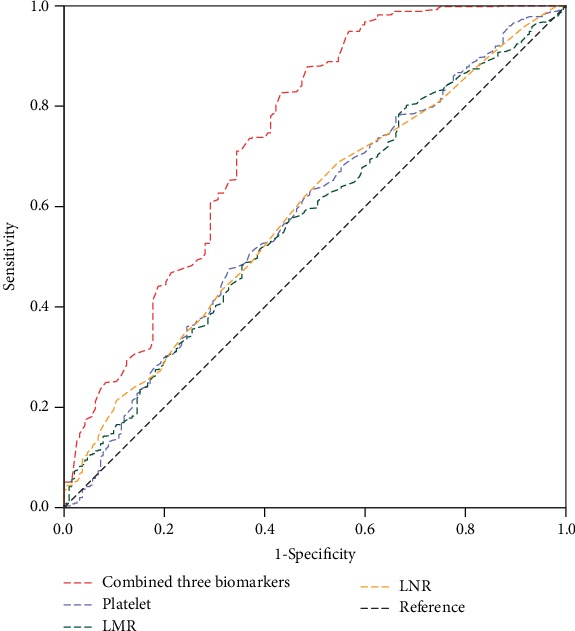
Receiver operating characteristic (ROC) curves of platelet counts, lymphocyte to neutrophil ratio (LNR), lymphocyte to monocyte ratio (LMR), and combined three biomarkers for 30-day mortality. The predictive probability of the ROC model was 0.739 (95% CI 0.695-0.784) when combined three biomarkers.

ROC curves for 30-day mortality for platelet counts, LNR, and LMR were shown in Figure 2. The c-statistics for platelet counts was 0.583 (95% confidence interval (CI) 0.536-0.630), with a cut point of 179.5 (sensitivity 48%, specificity 67%), for LNR was 0.589 (95% CI 0.543-0.635), with optimal cut point of 0.075 (sensitivity 69%, specificity 45%), and for LMR was 0.575 (95% CI 0.528-0.621), with a cut point of 1.435 (sensitivity 49%, specificity 64%). The correlation between platelet counts and LNR (*r* = 0.138, *p* < 0.001) and platelet counts and LMR (*r* = 0.074, *p* = 0.045) was very weak, while that between platelet counts and LMR (*r* = 0.001, *p* = 0.969) was no correlation.

The predictive value of each biomarker for 30-day mortality is shown in Table 2. Platelet counts, LNR, and LMR could predict 30-day mortality in univariate Cox regression models; however, they could not predict 30-day mortality in multivariate Cox regression models after adjusting other risk factors.

### 3.3. Combined Predictive Value of Three Biomarkers (Platelet Counts, LNR, and LMR)

**Figure 3:**
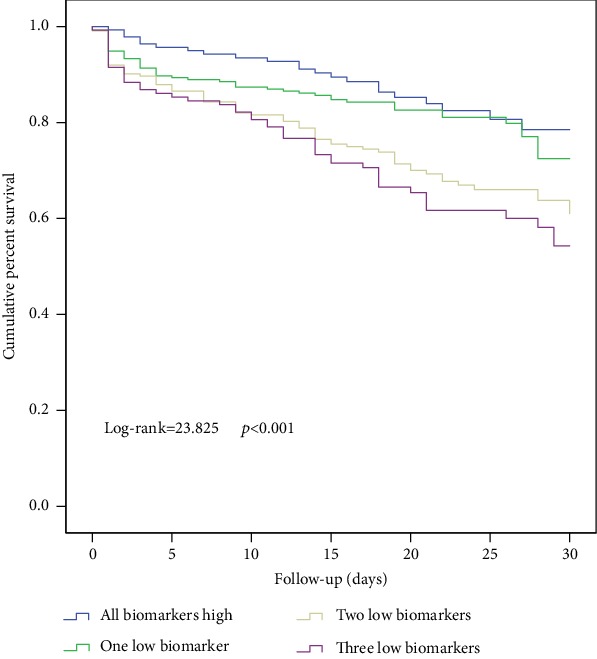
Kaplan-Meier survival of 30-day mortality for patients with zero, one, two, and three low biomarkers (log − rank = 23.825, *p* < 0.001). Patients with three low biomarkers had lower 30-day survival.

According to the number of biomarkers below the optimal cut-off values, the patients were divided into 4 groups: all biomarkers high (*n* = 139), one low biomarker (*n* = 253), two low biomarkers (*n* = 223), and three low biomarkers (*n* = 129), respectively. AAAD patients with all three biomarkers below the cut-off value had a higher risk of 30-day mortality (log-rank test *p* < 0.001) than AAAD patients with all three biomarkers above the cut-off value (Figure 3). The ROC model had a prediction probability of 0.739 (95% CI 0.695-0.784) when platelet counts, LNR, and LMR were included (Figure 2). After adjustment for other factors, patients with all three low biomarkers had significantly increased risk of 30-day mortality before (HR = 4.66, 95% CI 2.04-10.66, *p* < 0.001) or after (HR = 2.70, 95% CI 1.16-6.29, *p* = 0.021) surgical intervention was added to the Cox multivariate model (Table 2). Patients with all three low biomarkers had significantly higher rates of renal insufficiency (*p* < 0.001), liver insufficiency (*p* = 0.042), and gastrointestinal hemorrhage (*p* = 0.011), compared with patients with all three high biomarkers (Table 3).

## 4. Discussion

In this large sample cohort study of patients with AAAD, individual biomarker (platelet counts, LNR, or LMR) was unable to predict 30-day mortality. Moreover, combinations of all three biomarkers (platelet counts, LNR, and LMR) provided additive predictive value over either marker alone, which was the strongest predictor of 30-day mortality. In all AAAD patients, the calculation of these three biomarkers is easy to achieve without having to pay more for the patient or the medical system. This increases the potential value of the index.

An acute inflammatory response and platelet counts were known biomarkers associated with the development and outcome of AAAD [6, 13, 15–17]. There are no sensitive or specific diagnostic laboratory tests for AD. New tests that may confirm AD, such as those that measure soluble elastin fragments, smooth muscle myosin heavy chain, acute-phase reactants, such as the WBC count, high sensitivity CRP, fibrinogen, and D-dimer, are being developed [17]. These markers also exhibit different time courses of their changes in acute-phase reactions and between acute and chronic aortic diseases. More interestingly, they are associated with a poor prognosis and remain elevated even after repair of the dissection. Previous studies have focused on the prognostic value of individual indicators for AAAD, which is difficult to obtain an ideal prediction effect. New research suggests that AAAD is a disease that results from a combination of inflammation and blood clots [18]. Neutrophils, monocytes, and lymphocytes play important roles in the inflammatory state of AAAD, while platelets reflect the degree of thrombosis in AAAD [19–21]. Platelets play the role of “first stress response” in the pathogenesis of AAAD [22]. Platelet activation, aggregation, and release of inflammatory factors and growth factors into the vascular microenvironment can activate neutrophils and monocytes, promote lymphocyte migration to peripheral lymph nodes, and further promote platelet adhesion, aggregation, and thrombosis [23, 24]. The activation of neutrophils and endothelial cell adhesion to generate a large amount of reactive oxygen intermediate (ROI), in turn, increasing the vascular endothelial damage, and the excessive consumption of platelets after thrombosis may also increase the risk of aortic dissection rupture [25]. Therefore, inflammatory or thrombotic markers alone are not sufficient to reflect the whole pathophysiological process of AD. In contrast, inflammatory and thrombotic markers combined with intercellular signal transduction information may be better able to study the complete thromboinflammatory process in AAAD. Based on this mechanism, we have demonstrated that the coexistence of inflammatory biomarkers (LNR and LMR) and thrombotic marker (platelet counts) is a strong predictor of 30-day mortality than either of these biomarkers alone. Although previous studies also confirmed that high sensitivity CRP and D-dimer were associated with a poor prognosis in aortic dissection patients [26], these markers also exhibiting different time course of their changes in acute-phase reactions [27, 28], while three biomarkers that integrate multiple pathways of inflammatory and thrombotic processes, which may provide a better and more stable assessment of the prognosis of AAAD patients. Moreover, the three biomarkers were obtained from blood routine samples, which were of great significance for poor areas in terms of economy, when compared to other laboratory tests for AAAD.

Previous studies have demonstrated the neutrophil/lymphocyte ratio (NLR) to be a good predictor of in-hospital mortality [6, 11, 29]. In Karakoyun et al.'s study [11], they found that an NLR >8.51 yielded an AUC value of 0.829. In our study, however, LNR was not a significant independent predictor of 30-day mortality in AAAD patients, and the diagnostic value of an AUC could only reach 0.589. These differences may be explained by the following reasons. First, the time of LNR measurement is still unclear. Azab et al. [30] found that the average NLR during the whole hospital stay was the best in predicting patients' prognosis compared to the admission, discharge, or maximum NLR, while Park et al. [31] demonstrated that compared to admission NLR, 24-hour NLR was better at predicting mortality than the admission NLR. Second, the sample in Karakoyun et al.'s study was relatively small, only 35 patients with AAAD, for small sample size may exist sampling errors.

Our study also indicated that lower platelet counts were associated with 30-day mortality. Platelets not only participate in blood coagulation but also secrete a variety of cytokines that directly or indirectly participate in the body's inflammatory response. Huang et al. [5] reported that patients with admission platelet counts ≤119 × 109/L was an independent predictor of in-hospital death. In our study, the cut-off of platelet counts was 179.5 × 109/L, and patients with platelet counts less than 179.5 × 109/L are associated with 30-day mortality. However, platelet counts in our study was not an independent predictor of 30-day mortality after adjustment for other risk factors, this may be attributed to the different selective intercept values for platelet counts, the cut-off of platelet counts is higher than Huang et al.'s. Another important reason why platelet counts is not an independent prognostic in patients with AAAD may due to surgical intervention. In our center, we applied our open triple-branched stent-graft placement technique in AAAD patients [32], this surgical procedure had greatly shortened the cardiopulmonary bypass time, with an average of about 150 minutes. Extracorporeal circulation not only decreased the number of platelets and destroys the morphology of platelets but also affected the adhesion and aggregation of platelets. Several studies have shown that the duration of extracorporeal circulation was related to platelet counts and platelet function [33, 34]. In summary, improvements in the surgical procedure may lead to a good prognosis in some patients with lower platelet counts at admission.

We also observed that AAAD patients with all three low biomarkers had significantly higher rates of renal insufficiency, liver insufficiency, and gastrointestinal hemorrhage, compared with patients with all three high biomarkers. Although surgical treatment has an important impact on improving the prognosis of patients with AAAD, poor preoperative status also indicated the therapeutic effect of surgery to some extent [35]. All the three biomarkers were lower than the cut-off value, indicating that the AAAD patients had poor preoperative status. The lower platelet counts suggested platelet dysfunction, which increased the overall bleeding risk of perioperative patients and also aggravated the ischemia-reperfusion injury. Lower LNR and LMR suggested that patients are in a more severe inflammatory state before surgery, a large number of neutrophils and monocytes were activated, aggravating local inflammatory response, while lymphocytes represented the regulation of the immune system pathway, a decreased number of lymphocytes also indicated a poor outcome for cardiovascular disease [2].

There are some limitations in this study: (1) this is a retrospective study. In the future, the best critical point and normal range of each indicator should be further determined by large sample, multicenter, randomized controlled trials; (2) this study lack of measurement of C-reactive protein for C-reactive protein is not routinely tested in our study population for economic reasons; (3) in our study, we only evaluated the combined effects of triple risk factor, but could not evaluate the effect size of each risk factor. Future research can further explore the exact relationship between the three factors by experimental techniques.

## 5. Conclusion

In this relatively large cohort study, we found that combining three readily available admission biomarkers, namely platelet count, LNR, and LMR, helped identify AAAD patients with a high risk of 30-day mortality. All these biomarkers are available in blood routines, which greatly reduce the financial burden on AAAD patients.

## Figures and Tables

**Table 1:** Baseline characteristics of patients for 30-day mortality.

Variables	30-day mortality (*n* = 193)	30-day survivors (*n* = 551)	Hazard ratio (95% CI)	*p* value
Age (years)	58.5 ± 13.1	51.8 ± 11.6	1.04 (1.03-1.06)	<0.001
Male gender	140 (72.5)	431 (78.2)	0.73 (0.53-1.00)	0.051
BMI	24.70 ± 4.16	24.30 ± 3.77	1.027 (0.97-1.09)	0.350
Smoker	80 (41.4)	277 (50.2)	1.38 (1.04, 1.84)	0.028
Alcohol consumption	72 (37.3)	229 (41.5)	1.16 (0.86-1.55)	0.327
Diabetes mellitus	6 (3.1)	22 (3.9)	1.39 (0.62-3.13)	0.429
Hypertension	147 (76.1)	415 (75.3)	1.04 (0.75-1.45)	0.819
Marfan syndrome	4 (2.0)	13 (2.3)	1.08 (0.40-2.92)	0.874
SBP (mmHg)	137.8 ± 30.0	140.8 ± 28.7	1.00 (0.99-1.00)	0.166
DBP (mmHg)	76.6 ± 16.9	75.5 ± 16.4	1.00 (0.99-1.01)	0.584
PP (mmHg)	61.2 ± 22.2	65.4 ± 21.7	0.99 (0.98-1.00)	0.026
Heart rate (beats/min)	82.3 ± 16.8	82.3 ± 16.8	1.01 (1.00-1.02)	0.085
D-dimer (mg/L)	17.0 (6.7, 20.0)	10.9 (3.7, 20.0)	1.03 (1.00-1.05)	0.024
WBC	12.8 (10.0, 15.0)	11.7 (9.4, 14.3)	1.02 (0.98-1.06)	0.082
Serum creatinine (umol/L)	91.6 (71.4, 133.0)	85.8 (68.1, 123.0)	1.00 (1.00-1.00)	0.004
Total protein (g/dL)	62.7 ± 7.2	63.8 ± 6.9	0.98 (0.96-1.00)	0.055
Surgical intervention	101 (52.3)	549 (99.6)	0.04 (0.03-0.05)	<0.001

Values are reported as mean ± SD, *n* (%) or median (IQR). BMI: body mass index; SBP: systolic blood pressure; DBP: diastolic blood pressure; PP: pulse pressure; WBC: white blood cell.

**Table 2:** Predictive value of each biomarker and biomarker combination for 30-day mortality using univariate and multivariate Cox regression analysis.

	30-day mortality					
	Univariate analysis		Multivariate analysis^&^		Multivariate analysis^$^	
	Hazard ratio (95%)	*p* value	Hazard ratio (95%)	*p* value	Hazard ratio (95%)	*p* value
Platelet count	0.997 (0.995-1.000)	0.020	0.998 (0.995-1. 001)	0.190	0.999 (0.996-1.002)	0.512
LNR	0.12 (0.03,0.58)	0.008	0.02 (0.00-0.44)	0.012	0.11 (0.01-1.72)	0.115
LMR	0.83 (0.72,0.96)	0.010	0.80 (0.65-0.98)	0.033	0.95 (0.78-1.15)	0.572
One low biomarker	1.29 (0.78-2.13)	0.323	2.35 (1.07-5.16)	0.033	2.16 (0.98-4.77)	0.058
Two low biomarkers	2.15 (1.33-3.46)	0.002	3.16 (1.45-6.91)	0.004	1.70 (0.75-3.86)	0.203
Three low biomarkers	2.60 (1.57-4.30)	<0.001	4.66 (2.04-10.66)	<0.001	2.70 (1.16-6.29)	0.021

^&^adjusted for age, smoker, pulse pressure, serum creatinine, and D-dimer. ^$^adjusted for age, smoker, pulse pressure, serum creatinine, D-dimer, and surgical intervention. LNR: lymphocyte to neutrophil ratio; LMR: lymphocyte to monocyte ratio.

**Table 3:** 30-day in-hospital outcomes between the group of all biomarkers high and the group of three low biomarkers.

Variables	All biomarkers high (*n* = 139)	Three low biomarkers (*n* = 129)	*p* value
Renal insufficiency	6 (4.3)	25 (19.4)	<0.001
Liver insufficiency	6 (4.3)	14 (10.9)	0.042
Cerebral infarction	4 (2.9)	7 (5.4)	0.293
Arrhythmia	1 (0.7)	2 (1.6)	0.948
Gastrointestinal hemorrhage	3 (2.2)	12 (9.3)	0.011
Respiratory failure	0	1 (0.8)	0.970
MODS	3 (2.2)	6 (4.7)	0.428

MODS: multiple organ dysfunction syndrome.

## Data Availability

The data is in our database, readers can contact with us they need it.
